# Neuroimaging Studies in Patients With Mental Disorder and Co-occurring Substance Use Disorder: Summary of Findings

**DOI:** 10.3389/fpsyt.2019.00702

**Published:** 2019-10-23

**Authors:** Kaloyan Rumenov Stoychev

**Affiliations:** Department of Psychiatry, Medical University Pleven, Pleven, Bulgaria

**Keywords:** neuroimaging studies, comorbidity, substance use disorders, mood disorders, anxiety disorders

## Abstract

**Introduction:** More than half of psychiatric patients have comorbid substance use disorder (dual diagnosis) and this rate, confirmed by many epidemiological studies, is substantially higher compared to general population. Combined operation of self-medication mechanisms, common etiological factors, and mutually causative influences most likely accounts for comorbidity, which, despite its clinical prevalence, remains underrepresented in psychiatric research, especially in terms of neuroimaging. The current paper attempts to review and discuss all existing methodologically sustainable structural and functional neuroimaging studies in comorbid subjects published in the last 20 years.

**Methods:** Performing a systematic PubMed/MEDLINE, Web of Science, and Cochrane databases search with predefined key-words and selection criteria, 43 structural and functional neuroimaging studies were analyzed.

**Results:** Although markedly inconsistent and confounded by a variety of sources, available data suggest that structural brain changes are slightly more pronounced, yet not qualitatively different in comorbid patients compared to non-comorbid ones. In schizophrenia (SZ) patients, somewhat greater gray matter reduction is seen in cingulate cortex, dorsolateral prefrontal and frontotemporal cortex, limbic structures (hippocampus), and basal ganglia (striatum). The magnitude of structural changes is positively correlated to duration and severity of substance use, but it is important to note that at least in the beginning of the disease, dual diagnosis subjects tend to show less brain abnormalities and better cognitive functioning than pure SZ ones suggesting lower preexisting neuropathological burden. When analysing neuroimaging findings in SZ and bipolar disorder subjects, dorsolateral prefrontal, cingular, and insular cortex emerge as common affected areas in both groups which might indicate a shared endophenotypic (i.e., transdiagnostic) disruption of brain networks involved in executive functioning, emotional processing, and social cognition, rendering affected individuals susceptible to both mental disorder and substance misuse. In patients with anxiety disorders and substance misuse, a common neuroimaging finding is reduced volume of limbic structures (n. accumbens, hippocampus and amygdala). Whether this is a neuropathological marker of common predisposition to specific behavioral symptoms and drug addiction or a result from neuroadaptation changes secondary to substance misuse is unknown. Future neuroimaging studies with larger samples, longitudinal design, and genetic subtyping are warranted to enhance current knowledge on comorbidity.

## Introduction

The co-occurring mental disorder and substance use disorder (SUD), a phenomenon also referred to as comorbidity or the older term *dual diagnosis* (DD) (1, 2), has been consistently replicated in a number of large epidemiological studies in the last three decades (3–10). Over 50% of psychiatric inpatients have a co-occurring SUD (11), and this rate is far bigger than what is found in general population (12) and predicted by a mere coincidence model (13). On the other hand, more than half of the individuals diagnosed with SUD meet the criteria for another mental disorder, the most common ones being anxiety, mood, personality, and schizophrenia/psychotic disorders (11). Dual-diagnosis subjects impose a serious challenge because of the higher severity of medical problems, social and familial burden, and the greater incidence of relapses related to both mental disorder and SUD (14).

Despite being conceptually criticized on multiple levels (15), the invariable presence of comorbidity in everyday practice has invoked a number of explanation attempts (5, 16). They may be broadly subdivided into [1] illness-mediated theories—an index disorder causes the secondary/comorbid condition; [2] theories of common causal factors—one or more independent etiological factors increase the risk for both disorders; [3] bidirectional theories—presence of mutually reciprocal causal influences between the comorbid disorders. Most epidemiological studies indicate that in terms of occurrence, the mental disorder has a temporal priority (4, 5, 13, 17, 18), thus lending credibility to the so-called “self-medication” hypothesis (19, 20), considering SUD as a secondary result of repeating substance use in an attempt to alleviate mental disorder symptoms. It is much more likely, however, that a combination of mechanisms acts for each pattern of comorbidity in each particular patient—for example, self-medication and bidirectional mechanisms are implicated in anxiety disorders–SUD association (21, 22), while in patients with schizophrenia and SUD, common neurobiological, neurodevelopmental, and genetic causal factors are intertwined with self-medication mechanisms (20, 23, 24).

Because in the last several decades psychiatry has built diagnostic categories resting exclusively on clinical symptoms (25), most neuroimaging studies have focused on brain structure or function in patients with particular diagnosis, comparing them with healthy controls. As a result, proportionately few studies have included comorbid patients (26), and the vast majority of them focus on schizophrenia and co-occurring SUD (27). Furthermore, at least to our knowledge, there are no studies comparing groups of DD subjects with different disorders (e.g., depression vs. anxiety disorder). However, neuroimaging research suggests shared neurobiological abnormalities in phenotypically and genetically related diagnoses such as schizophrenia and bipolar disorder (BD) (28–30), and data also coalesce around the hypothesis that different psychiatric illnesses entail pertuberations along the same neural circuits (31, 32). Taking this into account, the current article aims to review and discuss reported data with some focus on the possible cross-diagnostic validity of findings.

## Methods

PubMed/MEDLINE, Web of Science, and Cochrane databases were searched with the following keywords and word combinations: “Co-occurring disorders,” “Comorbidity,” “Dual diagnosis,” “Magnetic resonance imaging” (MRI) and “functional Magnetic resonance imaging (fMRi),” “Schizophrenia and substance use disorder (SUD),” “Bipolar disorder and SUD,” “Depression and SUD,” “Anxiety disorder(s) and SUD”. Besides that, in the process of analysis of the initially chosen publications, all appropriate papers indexed in the reference sections were inspected. Previous reviews focusing on similar topics were also taken into consideration as a cross-reference.

Articles were selected according to the following criteria: *a*) dated between January 1999 and July 2019; *b*) written in English; *c*) published in full text; *d*) using widely recognized and popular neuroimaging technique, e.g., MRI, PET etc.; *e*) performed in humans; *f*) including subjects meeting *International Statistical Classification of Diseases,-10th Revision*/*Diagnostic and Statistical Manual of mental disorders, Fourth Edition* (Fifth) (*DSM-IV*(*5*)) criteria for abuse of or dependence on at least one of the following substances: alcohol, cannabinoids, cocaine, hallucinogens, medicinal drugs (e.g., benzodiazepines), opioids, and stimulants (amphetamines, ecstasy).

## Results

The initial search made 91 hits, of which 53 were excluded based on selection criteria. In the process of reviewing, five more relevant publications emerged from reference literature and were included. Finally, 43 studies were chosen for participation in this review, and they are summarized on **Tables 1**–**4**.

**Table 1:** Structural neuroimaging findings in first-episode or recent-onset schizophrenic patients with SUD (duration of symptoms <5 years).

Authors	Type of study and sample size	Study population/diagnoses	Results
Scheler-Gilkey et al. (36)	Cross-sectional, MRI (qualitative assessment); N = 176	Male and female adults; SZ/SAD + AUD and SZ/SAD + SUD (n = 103) vs. SZ/SAD only (n = 73)	No difference on brain MRI
Cahn et al. (37)	Cross-sectional, MRI (RoI, VBM); N = 47	Male and female adults; recent-onset SZ/SFD/SAD + lifetime CUD (n = 27) vs. recent-onset SZ/SFD/SAD (n = 20)	No difference in brain MRI; reduced asymmetry of the lateral ventricles in the SZ/SFD/SAD + CUD-subgroup
Joyal et al. (38)	Cross-sectional, MRI (RoI); N = 64	Male adults; SZ + AUD (n = 19) vs. SZ only (n = 19) vs. HC (n = 26)	Significantly lower volume of cerebellar vermis in patients compared to controls, most expressed in DD patients. Anomalies in the posterior vermis in both SZ groups and in anterior vermis only in DD group
Szeszko et al. (39)	Cross-sectional, MRI (RoI); N = 107	Male and female adults; first-episode SZ + CUD ± SUD (n = 20) vs. first-episode SZ only (n = 31) vs. HC (n = 56)	SZ + CUD ± SUD patients had less anterior cingulate GM compared with the other two groups
Bangalore et al. (40)	Cross-sectional, MRI (RoI, VBM); N = 81	Male and female adults; first-episode SZ/SAD/SFD + lifetime CUD ± SUD (n = 15) vs. first-episode SZ/SAD/SFD only (n = 24) vs. HC (n = 42)	More prominent GM density and volume reduction in the right PCC in DD patients compared to SZ only group
Rais et al. (41, 42)	Cross sectional, 5 y longitudinal, MRI (VBM); N = 82	Male and female adults; first-episode SZ + CUD (n = 19) vs. first-episode SZ only (n = 32) vs. HC (n = 31)	Larger GM volume loss, greater lateral ventricle enlargement and more pronounced cortical thinning in ACC and DLPFC in the SZ + CUD group compared to SZ only
Wobrock et al. (43)	Cross-sectional, MRI (RoI); N = 41	Male and female adults; recent-onset SZ/SAD + lifetime SUD (n = 20) vs. recent-onset SZ/SAD only (n = 21)	No differences in the assessed morphology (superior temporal gyrus, amygdala-hippocampus complex and cingulum)
Ebdrup et al. (44)	Cross-sectional MRI (VBM); N = 91	Male and female adults; first-episode SZ + lifetime SUD (n = 9) vs. first-episode SZ only (n = 29) vs. HC (n = 43)	Significant hippocampal and caudate volume reductions in both SZ groups. Decrease in hippocampal volume more pronounced in SZ + lifetime SUD
Ho et al. (45) and Onwuameze et al. (46)	Cross-sectional, MRI (RoI); N = 235	Male and female adults; SZ/SAD + CUD ± SUD (n = 52) vs. SZ/SAD ± SUD (n = 183)	SZ/SAD + CUD ± SUD had smaller frontotemporal WM volumes than SZ/SAD ± SUD. Significant genotype-by-cannabis use interaction effects on WM volumes and on neurocognitive impairment
James et al. (47)	Cross-sectional, MRI (DTI, VBM); N = 60	Male and female adolescents (13–18 y); recent-onset SZ + CUD (n = 16) vs. recent-onset SZ only (n = 16) vs. HC (n = 28)	Diffuse reduction in GM and WM in SZ patients compared to HC; greater GM density loss in DD group in temporal fusiform gyrus, parahippocampal gyrus, ventral striatum, right middle temporal gyrus, insular cortex, precuneus, right paracingular gyrus, DLPFC, left postcentral gyrus, lateral occipital cortex and cerebellum; greater WM loss in DD group in brainstem, internal capsule, corona radiata, superior and inferior longitudinal fasciculus
Cohen et al. (48)	Cross-sectional, MRI (VBM, cortical pattern matching); N = 55	Male and female adults; first-episode SZ + CUD (n = 6) vs. first-episode SZ only (n = 13) vs. CUD only (n = 17) vs. HC (n = 19)	SZ (with and without CUD) had lower total cerebellar GM than HC; no difference between SZ with and without CUD
Kumra et al. (49)	Cross-sectional, MRI (RoI); N = 115	Male and female adolescents; HC (n = 51) vs. CUD (n = 16) vs. early-onset SZ/SAD/SZF (n = 35) vs. early-onset SZ/SAD/SZF + CUD (n = 13)	Decreased GM volume in the left superior parietal cortex in all three patient groups compared to HC, least expressed in DD. The latter had less GM in the left thalamus, compared to CUD and SZ
Schnell et al. (50)	Cross-sectional, MRI (DORTEL-VBM, RoI); N = 54	Male and female adults; first-episode SZ + lifetime CUD (n = 30) vs. first-episode SZ only (n = 24)	Less severe middle frontal gray matter deficits as well as cognitive impairments in DD group
Cunha et al. (51)	Cross-sectional, MRI (VBM, RoI); N = 200	Male and female adults; first-episode psychosis-FEP (non-affective or affective) as assessed by SCID-IV; FEP + CUD (n = 28) vs. FEP only (n = 78) vs. HC (n = 94)	GM deficits in hippocampus and parahippocampal gyrus and PFC as well as LV enlargement in FEP only group compared to DD group; better cognitive performance of DD group (equal to HC)
Malchow et al. (52)	Cross-sectional, MRI (RoI); N = 79	Male and female adults; first-episode SZ + CUD (n = 29) vs. SZ only (n = 20) vs. HC (n = 30)	Decreased volume of the left hippocampus, bilateral amygdala and caudate nucleus and increased midsagittal CC1 segment of the corpus callosum in all SZ subjects. DD patients with family history of SZ showed lower volumes of the bilateral caudate nucleus and increased midsagittal area of the CC2 subsegment of the corpus callosum compared to all other patients
Epstein et al. (53)	Cross-sectional, MRI (RoI); N = 134	Male and female adolescents; HC (n = 53) vs. CUD (n = 29) vs. early-onset SZ/SAD/SFD (n = 34) vs. early-onset SZ/SAD/SFD + CUD (n = 18)	Smaller surface area in the right caudal ACC in DD group compared to SZ and CUD groups; this finding significantly correlates with less efficient executive attention
Koenders et al. (54)	Cross-sectional, MRI (RoI); N = 197	Male adults; recent-onset SZ + CUD (n = 80) vs. resent-onset SZ only (n = 33) vs. HC (n = 84)	All SZ subjects had smaller volumes of most brain regions (amygdala, putamen, insula, parahippocampus, and fusiform gyrus) than HC. SZ + CUD had a larger volume of the putamen compared to SZ only, possibly explained by polysubstance use\

RoI, region(s) of interest; VBM, voxel-based morphometry; DTI, diffusion tensor imaging; SZ, schizophrenia; SAD, schizoaffective disorder; SFD, schizophreniform disorder; AUD, alcohol use disorder (abuse or dependence); AD, alcohol dependence; SUD, substance use disorder (abuse or dependence) including the following substances (varying dependent on study): cocaine, stimulants (amphetamines, ecstasy), hallucinogens, opioids, cannabinoids, alcohol, medicinal drugs; CUD, cannabis use disorder (abuse or dependence); DD, dual diagnosis; HC, healthy controls; ACC, anterior cingulate cortex; PCC, posterior cingulate cortex; DLPFC, dorsolateral prefrontal cortex; VLPFC, ventrolateral prefrontal cortex; PFC, prefrontal cortex; LV, lateral ventricles; WM, white matter; GM, gray matter.

**Table 2:** Structural neuroimaging findings in schizophrenic patients with illness duration >5 years and comorbid SUD.

Authors	Type of study and sample size	Study population/diagnoses	Results
Sullivan et al. (55)	Cross-sectional, MRI (RoI); N = 132	Male adults; SZ only (n = 27) vs. SZ + AD (n = 19) vs. AD only (n = 25) vs. HC (n = 61)	Ventricular enlargement in both SZ groups, greater in DD patients. Decreased cerebellar volume in DD and AD patients, not in SZ only ones
Sullivan et al. (56)	Cross-sectional, MRI (RoI); N = 122	Male adults; SZ only (n = 27), Vs. SZ + AD (n = 19) vs. AD only (n = 25) vs. HC (n = 51)	Volume deficits in pons in DD patients
Mathalon et al. (57)	Cross-sectional, MRI (RoI); N = 223	Male adults; SZ/SAD + lifetime AUD (n =35) vs. SZ/SAD only (n = 64) vs. AD only (n = 62) vs. HC (n = 62)	Greatest GM volume deficits in DD group, particularly in the prefrontal and anterior superior temporal brain regions
Deshmukh et al. (58)	Cross-sectional, MRI (RoI); N = 122	Male adults; SZ only (n = 27) vs. SZ + AD (n = 19) vs. AD only (n = 25) vs. + HC (n = 51)	Caudate, putamen, and nucleus accumbens showed different patterns of volume deficits in SZ and AD; no evidence for compounded deficits in DD group
Potvin et al. (59)	Cross-sectional, MRI (VBM); N = 38	Male and female adults; SZ + AUD/CUD (n = 12) vs. SZ only (n = 11) vs. HC (n = 15)	Increased gray matter density in the ventral striatum in DD compared with pure SZ
Schiffer et al. (60)	Cross-sectional, MRI (VBM); N = 51	Male adults; SZ + current or lifetime SUD (n = 12) vs. SZ only (n = 12) vs. SUD only (n = 13) vs. HC (n = 14)	GM matter losses in lateral orbitofrontal and temporal regions associated with SZ, and in medial orbitofrontal, ACC and frontopolar cortex with addiction. DD subjects had higher volume decreases in ACC, frontopolar and superior parietal regions and increased nonplanning impulsivity compared to SZ
Smith et al. (61)	Cross-sectional, MRI (HDBM-LD); N = 107	Male and female adults; SZ + past AUD (n = 16) vs. SZ only (n = 35) vs. HC (n = 56)	DD group had more severe shape abnormalities in the hippocampus, thalamus, striatum, and globus pallidus compared to SZ only group
Solowij et al. (62, 63),	Cross-sectional, MRI (RoI, semiautomatic method); N = 48	Male adults; SZ + CUD (n = 8) vs. SZ only (n = 9) vs. CUD only (n = 15) vs. HC (n = 16)	Significantly smaller cerebellar WM volume and hippocampal shape change in all patient groups compared to HC, most severely expressed in DD group
Gizewski et al. (64)	Cross-sectional, MRI (VBM); N = 48	Male adults; HC (n = 12) vs. AD (n = 12) vs. SZ + AD (n = 12) vs. SZ only (n = 12)	All SZ patients (AD and non-AD) had reduced GM volume in the left VLPFC compared to HC
Smith et al. (65)	Cross-sectional, MRI (HDBM-LD); N = 97	Male and female adults; HC (n = 44) vs. past CUD (n = 10) vs. SZ only (n = 28) vs. SZ + past CUD (n = 15)	Similar cannabis related shape differences (suggestive of localized volume loss) in the striatum, globus pallidus, and thalamus in past CUD and SZ + past CUD more pronounced in the latter. Significant cannabis related decrease in working memory across groups

RoI, region(s) of interest; VBM, voxel-based morphometry; HDBM-LD, large-deformation high-dimensional brain mapping; SZ, schizophrenia; SAD, schizoaffective disorder; SFD, schizophreniform disorder; AUD, alcohol use disorder (abuse or dependence); AD, alcohol dependence; SUD, substance use disorder (abuse or dependence) including the following substances (varying dependent on study): cocaine, stimulants (amphetamines, ecstasy), hallucinogens, opioids, cannabinoids, alcohol, medicinal drugs; CUD, cannabis use disorder (abuse or dependence); DD, dual diagnosis; HC, healthy controls; ACC, anterior cingulate cortex; PCC, posterior cingulate cortex; DLPFC, dorsolateral prefrontal cortex; VLPFC, ventrolateral prefrontal cortex; PFC, prefrontal cortex; LV, lateral ventricles; WM, white matter; GM, gray matter.

**Table 3:** Functional neuroimaging findings in schizophrenic patients with SUD.

Author	Type of study and sample size	Study population/diagnoses	Results
Mancini-Marïe et al. (66)	Functional MRI (fMRI) during passive viewing of emotionally negative pictures; N = 23	Adults; SZ + SUD (n = 12) vs. SZ only (n = 11)	Heightened activity in the right medial prefrontal cortex, left medial prefrontal cortex, right orbitofrontal cortex, and left amygdala only in DD group who also showed higher subjectively rated experience
Joyal et al. (67)	fMRI during execution of a go/no-go task measuring response inhibition capacity; N = 36	Adult males; homicide offenders with SZ + APD + SUD (n = 12) vs, SZ only (n = 12) vs. HC (n = 12)	Substantially less activation of frontal basal cortices and higher activation in frontal motor, premotor and anterior cingulate regions in SZ + APD + SUD group compared to the other two groups; findings related to personality characteristics (antisocial behavior) and not to SZ
Potvin et al. (68)	fMRI during passive viewing of an emotional film excerpt with social content; N = 22	Adult males and females; SZ + SUD (AUD/CUD/AUD + CUD) in the last 18 mo (n = 11) vs. SZ only (n = 11).	Increased activation in right superior parietal cortex and left medial prefrontal cortex in DD patients in comparison to SZ only group. The former also had higher subjective emotional experience on a self-report scale.
Løberg et al. (69)	fMRI during auditory listening task engaging verbal processing, attention and cognitive control; N = 26	Adult males and females; SZ + CUD (n = 13) vs. SZ only (n = 13)	Very similar activation patterns of both groups overall; slight difference in cortical activation dynamics of the default mode network and cognitive performance in favor of the SZ + CUD group
Bourque et al. (70)	fMRI during task for encoding and recognition of a series of positive and negative pictures; N = 49	Male adults; SZ + CUD (n = 14) vs. SZ only (n = 14) vs. HC (n = 21)	Recognition of positive and negative stimuli prominently impaired in SZ group compared to DD and HC. Emotional memory and prefrontal lobe functions preserved in DD in comparison to SZ patients
Thompson et al. (71)	[^11^C] raclopride PET in two sessions: baseline and after receiving amphetamine; N = 26	Male and female adults; SZ/SAD + SUD (n = 11) vs. HC (n = 15)	DD subjects displayed significant blunting of striatal DA release suggesting that in SUD-SZ patients, hypersensitivity of D2 receptors rather than excess presynaptic dopamine release is the predominant dopaminergic alteration
Gizewski et al. (64)	fMRI with mind reading task that involves empathy (cognitive and affective); N = 48	Male adults; HC (n = 12) vs. AD (n = 12) vs. SZ + AD (n = 12) vs. SZ only (n = 12)	All SZ patients (AD and non-AD) had decreased activity in left VLPFC; all clinical groups (as opposed to HC) had decreased activity in AIC; DD patients had more preserved social skills compared to SZ only patients

SZ, schizophrenia; APD, antisocial personality disorder; AUD, alcohol use disorder (abuse or dependence); CUD, cannabis use disorder; SUD, substance use disorder (abuse or dependence) including the following substances: cocaine, stimulants (amphetamines, ecstasy), cannabinoids, alcohol; HC, healthy controls; DD, dual diagnosis; VLPFC, ventrolateral prefrontal cortex; AIC, anterior insular cortex.

**Table 4:** Structural and functional neuroimaging findings in studies with nonschizophrenic patients with SUD.

Author	Type of study and sample size	Study population/diagnoses	Results
De Bellis et al. (72)	Cross-sectional, MRI (RoI); N = 42	Male and females adolescents and young adults (13–21 y); AUD + mental disorder* (n = 14) vs. HC (n = 28)	DD subjects had smaller PFC and PFC WM volumes compared with controls; significant sex-by-group effect in favor of males; PFC volume variables significantly correlated with measures of alcohol consumption
Woodward et al. (73)	Cross-sectional, MRI (RoI); N = 99	Male adults combat veterans (n = 99); PTSD + lifetime AUD vs. PTSD only vs. lifetime AUD only vs. HC	Smaller hippocampal volume in PTSD subjects more pronounced in DD group. PTSD was strongly associated with comorbid major depression
Hassel et al. (74)	fMRI with viewing of event-related paradigms of happy and fear faces; N = 30	Male and female adults: BD + SUD ± ED (n = 8) vs. BD only (n = 6) vs. HC (n = 16)	Reduced dorsal prefrontal-cortical activity to all faces and greater subcortical-striatal activity to happy and neutral faces in BD patients compared to controls; differences more expressed in DD group
Cornelius et al. (75)	BOLD fMRI using threat related amygdala reactivity paradigm; N = 6	Male and female adults; current CUD + current MDD (n = 6)	Amygdala reactivity inversely related to level of cannabis use suggesting an inhibitory effect of cannabinoids on amygdala function
Sameti et al. (76)	Cross-sectional, MRI (RoI, model-based segmentation PC software); N = 100	Male and female adults; past AUD + current or past mental disorder* (n = 52) vs. control group with past or current mental disorder (n = 48)	Minimal differences between groups in subcortical volumes (LV, thalamus, caudate, pallidum, putamen, hippocampus, amygdala, n. accumbens); in DD group, however, subcortical structures were smaller in those with vs. without current or past mental disorder
Nery et al. (77)	Cross-sectional, MRI (VBM); N = 67	Male and female adults; BD + past AUD (n = 21) vs. BD only (n = 21) vs. healthy controls (n = 25)	DD patients had smaller GM volumes in the left medial frontal and right anterior cingulate gyri compared to BD only group. The latter did not present GM volume differences compared to HC
Lippard et al. (78)	Longitudinal MRI, (VBM) with mean follow-up of 6 y; N = 30	Male and female adolescents and young adults with BD (n = 30)	Lower GM volume in prefrontal, insular, and temporopolar cortices were observed at baseline among adolescents with BD reporting subsequent alcohol and cannabis misuse compared to adolescents with BD who did not

*Mental disorder includes the following categories ranged according to the rate of occurrence: other substance abuse or dependence, major depressive disorder, conduct disorder, posttraumatic stress disorder, attention-deficit hyperactivity disorder, oppositional defiant disorder, generalized anxiety disorder, bipolar disorder, and antisocial personality disorder.

Legend: RoI = region(s) of interest; VBM, voxel-based morphometry; PFC, prefrontal cortex; WM, white (cerebral) matter; LV, lateral ventricles; PTSD, posttraumatic stress disorder; MDD, major depressive disorder; AUD, alcohol use disorder (abuse or dependence); BD, bipolar disorder; DD, dual diagnosis; GM, gray matter; WM, white matter.

### Schizophrenia and SUD

Schizophrenia is by far the most prevalent diagnosis in neuroimaging research on comorbidity and with regards to type of substance misuse, the majority of studies have enrolled patients with alcohol, cannabis, or multiple SUDs abuse or dependence (26, 33, 34). As for the investigational tools, all but one of the studies employ MRI (VBM, RoI, DTI) and fMRI. For perspicuity reasons, structural and functional neuroimaging studies are presented on separate tables in this review. In addition, based on the duration of illness and the age of the included population, structural neuroimaging studies for schizophrenia are subdivided into two separate tables: **Table 1** for studies including adolescent and young adult subjects with first episode or recent onset of the disease (up to 5 years) and **Table 2** for subjects with chronic schizophrenia lasting more than 5 years. This distinction was made for two reasons: first, this time interval was used in studies that include follow-up of patients with first-episode psychosis (41, 42). Second, studies of first-episode or recent-onset schizophrenia often include minimally treated or medication-naive subjects, which allows for better discrimination between structural changes imposed by substance use and those related to long-term antipsychotic treatment (35).

### Other Mental Disorders

Only a few studies have included comorbid subjects with diagnosis other than schizophrenia (**Table 3**), and these are predominantly mood disorders (BD and major depression), anxiety and stress-related disorders, and also conditions typically occurring in childhood or adolescence. In terms of visualization method, five of the studies use structural MRI, and two are fMRI studies.

## Discussion

The current review tries to summarize and interprete the results of all methodologically consistent neuroimaging studies that have focused on DD patients and have been carried out over the past 20 years. Prior to discussing their findings and suggesting possible implications, several essential considerations have to be emphasized.

First, the most substantial limitations of all reviewed studies examining comorbid subjects are small sample sizes. With the exception of a few studies [e.g., (36, 45–46, 51, 54, 73)], most authors have included 8 to 25 comorbid patients in their samples with a corresponding number of controls. Furthermore, some authors have employed the same or significantly overlapping sample for several different articles (55–58, 62–63, 69) or used one sample for a structural and a functional neuroimaging study (64). As a consequence, the number of examined comorbid patients is highly insufficient to allow any definite conclusions, given the discrete size differences in compared anatomical structures. Such an inference is even truer for comorbid disorders other than schizophrenia, which are extremely underrepresented in the literature. Hence, future studies with much larger samples and more diverse diagnostic categories are warranted to confirm or reject the findings reported so far.

An additional holdback to data reliability is the low geographic, racial, and ethnocultural variety of reported studies—with only one exception of a study from Brazil (51), all the rest originate from Western Europe (37, 38, 41–47, 50, 52, 54, 60, 64, 67, 69), USA and Canada (36, 39, 40, 49, 53, 55–58, 61, 65, 66, 68, 70–78), and Australia (62, 63), with no studies from Asia, Africa, and most other parts of Europe and South America. Moreover, with regards to structural neuroimaging research in schizophrenia, there is an overall sex inequality of the examined populations with males constituting at least two-thirds of the DD subjects in more than half of the studies (36, 37, 39, 43–48, 50, 52–54, 61, 65) and representing the only studied group in the rest (38, 55–58, 62–64). While reflecting the clinicoepidemiological reality of more common SUDs in men including those with a co-occurring mental illness (79), this fact further obstructs the possibility of making definite conclusions given the existing evidence for gender influence on brain morphology in schizophrenia (80). Another restriction of the available data concerns the adolescent population with co-occurring SUD and schizophrenia. Apart from the few conducted studies involving very small patient groups with cannabis misuse only (47, 49, 53), some of the published articles do not even include patients meeting the threshold criteria for abuse and/or dependence (81, 82) and for this reason have not been taken into consideration in the present review. This fact renders any sound inferences regarding structural and functional brain changes in adolescent DD patients even more problematic than those for adults.

One very important consideration concerns diagnostic heterogeneity in the studied samples. Despite the strictly defined study protocols employed by authors striving to include “pure” DD subjects in their samples, there is still marked heterogeneity in both the substance use disorder and the co-occurring mental disorder. Some of the structural neuroimaging studies including those with largest samples have enrolled patients with polysubstance misuse (36, 39, 40, 43–46, 60), and even those focusing on a specific SUD—largely cannabis—feature subjects with additional current or past substance use disorder, most often alcohol or stimulants misuse [e.g (51, 54, 69)]. Combined drug use is an important confounding factor, which, according to some of the authors, may explain certain structural differences found in DD subjects such as striatal volume differences (54, 60). Furthermore, one of the major studies in terms of sample size compares schizophrenic patients with cannabis misuse against other substance misusing (e.g., not “clear”) schizophrenic patients (45, 46). In one big study (54), the possible confounding effects of smoking, which has been reported in association with additional volume loss in schizophrenia (83), could not be separated, and this flaw is most likely also true for all the studies with adult SZ patients, although not explicitly stated by authors. As a result, the observed differences in brain morphology between comorbid and noncomorbid subjects could not be undoubtedly ascribed to the effects of a particular substance.

Substantial sample heterogeneity also exists for the co-occurring mental disorder. In fact, nearly all studies include patients with schizoaffective disorder (SAD) along with schizophrenics (SZ) in their samples (36, 37, 40, 43, 45, 46, 49, 53, 71). This shortcoming, resulting from both the inherent vagueness of SAD diagnosis and the categorical approach endorsed by structured diagnostic interviews (84), may as well bring some potentially positive implications. Specifically, considering the low clinical utility and reliability of SAD diagnostic criteria (85), and the low temporal consistency of SAD diagnosis found in longitudinal studies (86, 87), it could be hypothesized that the reported neuroimaging findings for comorbid SAD patients also apply to bipolar patients with a co-occurring SUD. Furthermore, one study has even included *DSM-IV*–defined “affective psychosis” subjects, and this category is largely limited to bipolar patients (51). Of course, given the scarce neuroimaging investigation focusing solely on bipolar comorbid subjects, future studies with sufficient magnitude are needed to test the validity of this assumption.

Another major drawback that should be outlined is related to the status of substance use disorders in the studied patients—particularly whether these are lifetime diagnoses in patients currently in stable and long-term remission or active phase conditions. In this line of thought, while some studies with comorbid schizophrenia and co-occurring SUD, particularly alcohol and cannabis, have only included subjects with past abuse or dependence [e.g., (40, 59, 60, 62, 63, 69)], others have investigated patients with a short or even no prior remission [e.g., (37, 54, 58)]. This might represent a substantial confounding factor since a positive correlation between recency of substance use (especially alcohol) and greater volume deficit in some brain structures such as nucleus accumbens has been reported (57).

The last constraint discussed here concerns concomitant antipsychotic treatment, which has a definite correlation with certain structural effects on the brain (35, 88). As most of the SZ study samples have been exposed to this class of medications [e.g., Refs. (41, 42, 45, 46, 54–60, 69) and others], its effects must necessarily be taken into account when discussing the results. Moreover, some authors (45–47) have found more structural changes in SZ patients with long-term therapy such as dysmorphology and volume deficits in the thalamus and striatal and other basal ganglia, more pronounced in those treated with atypical antipsychotics. Antipsychotics-related overall decrease in total brain gray and white matter has also been reported (45, 46). Taken together, these data further limit the significance and validity of reported neuroimaging findings in DD and non-DD subjects.

With all the considerations emphasized so far, the results of structural and functional neuroimaging studies in DD patients will be analyzed below as follows:

### Structural and Functional Neuroimaging Findings in Schizophrenia and SUD

As mentioned earlier, most of the studies have included predominantly cannabis, alcohol, and combined misuse subjects, and there is marked inconsistency across study findings, especially for cannabis use disorders (54). Whereas some studies detect no or insignificant differences between DD and non-DD patients (37, 43, 48, 58, 64), others suggest more severe structural changes in DD compared to non-DD patients (39–42, 44-47, 52, 60–63), and finally some authors find the opposite correlation (50, 51, 54). With regard to recent-onset or first-episode schizophrenia studies (i.e., with illness duration up to 5 years), reported findings consolidate around greater volume reduction on the expense of gray matter in DD subjects, with most commonly affected areas being anterior and posterior cingulate cortex (ACC, PCC) (39–42, 53), dorsolateral prefrontal cortex (DLPFC) (41, 42, 47), hippocampus (44, 52), striatum (47, 52), and frontotemporal cortex and cerebellum (39–42, 47). In addition, less cerebral white matter in frontotemporal cortex (45, 46) as well as diffuse subcortical areas (47) has been reported. None of these findings, however, is specific, and they represent only quantitative difference as compared to pure schizophrenia. Expectedly, in some studies, the magnitude of structural disruptions is positively correlated with neurocognitive impairment (45, 46, 53). Studies with chronic patients (i.e., with illness duration >5 years) also show slightly more pronounced volume loss in DD subjects affecting prefrontal, frontotemporal, parietal, and anterior cingulate cortical areas (57, 60), hippocampus (61–63), thalamus, striatum and globus pallidus (61, 65), cerebellar gray and white matter (55, 62, 63), and pons (56). Again, no specificity of findings may be claimed as the same are often reported in literature both with regard to pure schizophrenia (89) and pure alcohol and cannabis misuse (90, 91). The most distinctive neurocognitive impairment pattern in this group of studies was reported by Schiffer et al. (2010) who found significantly greater impulsivity in DD subjects compared to pure SZ ones, while executive functioning deficits of both groups were on par.

As noted above, studies that fail to demonstrate differences between DD and non-DD groups also exist. For example, with respect to alcohol use disorders (AUDs), Gizewski et al. (2013) found similar gray matter volume decrease in the left VLPFC in long-term abstinent alcoholic and nonalcoholic SZ groups compared to healthy controls. Deshmukh et al. (2005) comparing pure chronic SZ patients versus chronic alcohol-dependent SZ ones with a varying duration of preceding abstinence demonstrated somewhat greater volume deficit in SZ in striatum (putamen) and n. accumbens with no evidence for a compounded structural deficit in DD subjects (58). In the same sample, however, other authors did demonstrate greater ventricular enlargement and cerebellar volume loss (55) and more pronounced gray matter deficits in prefrontal and superior temporal cortex (57) and pons (56) in DD group, with the latter structure not considered as directly affected by schizophrenia (26). In a mixed SUD sample of recent-onset schizophrenics, Wobrock et al. (2009) found no differences in superior temporal gyrus, amygdala-hippocampus complex, and cingulum between comorbid and noncomorbid subjects (43). In a similar group of SZ patients with and without co-occurring alcohol or cannabis use disorder, Potvin et al. (2007) also did not find significant structural brain differences between groups except for increased gray matter density in the ventral striatum for the DD group (59). Interestingly, a similar finding of increased dorsal striatum (putamen) was reported by Koenders et al. (2009) in a comparison of cannabis misuse versus noncannabis misuse schizophrenic patients (54). As deficits in other areas (limbic structures, anterior cingulate, orbitofrontal and fusiform gyrus, insula, thalamus, and caudate) were not detected, the findings of both studies could be related to striatal neuroadaptation changes emerging from repetitive drug use (54, 59). Further considering cannabis use disorders, Cohen et al. (2012) did not detect differences in comorbid and noncomorbid subjects in a first-episode schizophrenia sample in which both groups had lower total cerebellar gray matter than healthy controls (48). Similarly, investigating a group of adolescent early-onset schizophrenia subjects with and without co-occurring cannabis use disorder, Kumra et al. (2012) did not find additional volumetric deficit in DD patients compared to pure EOS and pure CUDs, while all three groups had smaller gray matter volume in the left parietal cortex than controls. DD patients had somewhat smaller left hypothalamus than pure SZ subjects, but for the left parietal cortical surface, the opposite relationship was observed, and it was the size of this area that showed significant positive association with results on a neurocognitive test for attention and working memory supporting its stronger implication in schizophrenia-related neuropathological processes (49).

Finally, studies that favor DD subjects in terms of severity of illness and associated neuroimaging findings will be discussed. In the first of them, with stringent inclusion criteria regarding schizophrenia, Schnell et al. (2012) found less severe gray matter deficits in the left DLPFC in first-episode patients with past cannabis use disorder versus pure schizophrenia ones. Moreover, this result was paired with superior cognitive performance in verbal and working memory tests in the DD group (50). Similarly, in a larger study employing less rigorous selection criteria and thus including both nonaffective- and affective-type first-episode psychosis, Cunha et al. (2012) reported milder gray matter deficits in hippocampus, parahippocampal gyrus, and prefrontal cortex; smaller lateral ventricles enlargement and better cognitive performance in patients with cannabis use disorders versus those without (51). These results are in striking controversy with data from Rais et al. (41, 42), Ho et al. (45) and Onwuameze et al. (46) reported above, which indicate both more significant gray and white matter deficits in a number of cortical structures and worse performance on neurocognitive tests in cannabis misusing SZ subjects. In fact, explanations exist for both adverse and beneficial effects on marijuana in schizophrenia population. Regarding the former, theories suggest neurotoxic effects of cannabis, which are either direct *via* disturbed control of the endogenous cannabinoid system on glutamate and γ-aminobutyric acid release and subsequent impairment in maturation of neural circuitries in adolescence (92) or an indirect including complex genotype-by-cannabis interactions that leads to brain morphologic changes. Supporting that, in the only study of its type, Ho et al. (45) found significant association between more severe frontotemporal white matter deficits in DD subjects and a particular genetic variant of the cannabinoid 1 receptor (CNR1). The alternative set of explanations generally regards DD patients as a specific subgroup that is intrinsically less vulnerable to schizophrenia than cannabis-naive patients, has better premorbid cognitive functions and social adjustment (93), and probably would not have developed psychotic symptoms without the effects of substance use. Such a hypothesis regarding not only marijuana, but SUDs in general, is supported by the available functional neuroimaging research. Nearly all studies of this type report better preserved functioning in areas associated with emotional processing—medial prefrontal cortex (66, 68, 70) and social cognition—ventrolateral prefrontal cortex and anterior insular cortex (64). In addition, data show that areas associated with verbal processing and attention (posterior cingulate cortex, inferior parietal lobe and precentral gyrus) and executive functioning (DLPFC) also show higher activity in DD patients (69, 70). In further support of the hypothesis that comorbid SZ subjects might represent a subgroup with less neurobiological abnormalities than noncomorbid SZ, Thompson et al. (71) in a recent [^11^C] raclopride study hypothesized that a hypersensitivity of D2 receptors rather than excess presynaptic dopamine release is the predominant dopaminergic alteration in comorbid subjects. However, at least some preexisting neuropathological diathesis is seemingly necessary to reach psychotic state as witnessed by the study of Uhlmann et al. (2016) showing thinner prefrontal and temporal cortical areas and decreased hippocampal volume in methamphetamine-dependent patients with psychosis versus those without (94). In fact, both sets of explanations are not necessarily mutually exclusive: as hypothesized by Cunha et al. (51), the exposure to cannabis or other substances may be a prerequisite for development of first episode of psychosis in an initially relatively “preserved” brain, but with repeating use, severe gray matter deficit occurs, which is accountable for worse clinical and cognitive presentations of dual-diagnosis patients reported in longitudinal studies.

### Structural and Functional Neuroimaging in Patients With Diagnoses Other Than Schizophrenia

Although far more limited as compared to the comorbid schizophrenia research, available data are consistent with more severe neuroimaging changes in this population. Starting with BD, two structural and one fMRI studies demonstrate certain differences between comorbid and noncomorbid subjects. In the earliest of them, Hassel et al. (2009) showed abnormal pattern of brain activation in a small group of bipolar patients (n = 14) compared with controls (74). Using an event-related fMRI paradigm with happy, neutral, and sad faces, they found reduced dorsal prefrontal-cortical activity to all faces and greater subcortical-striatal activity to happy and neutral faces in all bipolar patients. Interestingly, decrease in prefrontal activity was more pronounced in comorbid patients, and authors have hypothesized that this phenomenon may be linked to stronger difficulties in integrating socioemotional information and, subsequently, emotion regulation. Moreover, similarly decreased DLPFC activity has been reported in substance abusers in decision making and facial matching tasks (95, 96). In a subsequent cross-sectional MRI study, Nery et al. (2011) found smaller gray matter volumes in the left medial frontal and right anterior cingulate gyri in a sample of bipolar patients with long-term remission AUD compared to pure bipolar ones (77). As these frontal lobe subareas are connected with other prefrontal areas and high-order association regions (orbitofrontal cortex, temporal and parietal lobe, and subcortical structures) and insofar prefrontal cortex plays an important role in the inhibitory control of inappropriate compulsive behaviors such as addiction (97), the authors suggested that the observed gray matter deficits are a structural correlate of the impaired “top-down” inhibitory control in prefrontal brain areas that distinguishes BD-AUD patients from BD patients without AUD (77). Supporting this assumption, in a recent study with longitudinal design, Lippard at al. (2017) reported lower baseline gray matter volume in prefrontal, insular, and temporopolar cortices in those adolescents who later developed alcohol and cannabis misuse, suggesting a possible endophenotype significance of these findings (78). Interestingly, sex-based difference in structural findings was also observed in that while decreased baseline gray matter volume in DLPFC was positively correlated with substance use problems in both females and males, lower orbitofrontal cortex and insular gray matter predicted substance use problems in females, whereas in males, these were associated with lower right prefrontal cortex gray matter volume. In addition to that, greater depressive symptoms at baseline were associated with greater substance use problems at follow-up, and depressive symptoms in females in particular were related to lower insular gray matter volume. Besides having a potential structural biomarker implication, this latter finding may also aid to see the popular explanatory theory of depression-SUD association as a manifestation of shared neurobiological vulnerability (98) in a new light. Further focusing on sex differences in brain structure, De Bellis et al. (72) in a sample of adolescents and young adult patients with alcohol and polysubstance misuse and an array of comorbid psychiatric diagnoses [i.e., mood, anxiety, and stress-related disorders; attention-deficit/hyperactivity disorder (ADHD); conduct and oppositional defiant disorders; and antisocial personality disorder] found smaller cerebellar gray matter volumes only in males. However, as this finding correlated substantially with a co-occurring diagnosis of ADHD, it was not regarded as associated with substance use. Such an association was found in fact, but with decreased gray and white matter volumes in prefrontal cortex, which was present in both sexes. The authors hypothesized that this finding might be either the result of direct or indirect detrimental effects of the substances on PFC development, a neurotoxic interference of the same with its maturation, or, alternatively, a reflection of inherent vulnerability for delayed PFC maturation subsequently enhancing the risk for poorer cognitive functioning and greater impulsivity and hence onset of substance misuse (72).

Other than BD, posttraumatic stress disorder (PTSD) has been most studied in nonpsychotic spectrum dual-diagnosis population. In a large study with 99 PTSD war veterans, a significant proportion of which had also comorbid depression, Woodward et al. (2006) found that past alcohol abuse or dependence has a significant inverse correlation with hippocampal volume (73). Although in nonalcoholic PTSD subjects the size of this structure was also reduced, the magnitude of the structural change was much smaller, suggesting that lower hippocampal volume might be a structural marker of shared neurobiological or genetic vulnerability to both alcoholism and PTSD (73). Further support for the close association between stress responses, limbic structures activity, and psychoactive substances was found in fMRI study by Cornelius et al. (2010) who investigated a small group of patients with comorbid cannabis dependence and depression (75). By means of threat-related amygdala reactivity paradigm, they showed that this structure known for its leading role in physiological and behavioral responses to stress and rich in CB1 receptors (99) displays reduced reactivity consistently correlating with the level of cannabis use. Such a finding supports the self-medication explanation theory for anxiety and substance use disorders comorbidity presented earlier. Additional evidence for implication of comorbid mood and anxiety disorders in structural brain changes in AUDs was presented by Sameti et al. (2011). By means of structural MRI comparison, these authors found in long-term abstinent alcoholics (LTAAs) smaller nucleus accumbens and hippocampus volumes in those LTAA individuals with a lifetime anxiety disorder than in those without (76). In addition to reduced n. accumbens, in alcohol-misusing patients with current anxiety disorder, a trend toward smaller putamen volumes was observed. Notably, the same association of smaller hippocampus and amygdala volumes in LTAA was also detected in subjects with a lifetime externalizing disorder diagnosis (i.e., conduct disorder, defiant disorder, ADHD, and antisocial personality disorder). The authors hypothesized that both internalizing (i.e., mood and anxiety) and externalizing disorders are associated with disrupted hypothalamic-pituitary-adrenal (HPA) axis response to stress and with impaired interactions of the former with mesolimbic reward circuitry, but this deviation is a result of two opposite mechanisms—a hypersensitization of the HPA axis with subsequent neurotoxic hypercorticism in mood/anxiety disorders and an undersensitization with hypocorticism in externalizing disorders (76). As a consequence, vulnerability to abuse of drugs is increased in both groups—in an attempt to reduce negative psychological effects of stress in mood/anxiety disorders and as a way of stimulating reduced HPA reactivity in externalizing disorders (similar to thrill and adventure-seeking behavior typical for this group of subjects).

## Conclusion

Definite conclusions would be substantially enhanced by future studies on comorbidity engaging much larger samples, endorsing more powerful longitudinal design, and enhanced by genetic polymorphism subtyping. Currently available data, although markedly inconsistent and confounded by a variety of sources (e.g., different study design, small and heterogenic samples, concomitant medications, smoking, etc.), support the assumption that in substance-misusing psychiatric patients structural brain changes are more pronounced, yet not qualitatively different from what is seen in noncomorbid subjects with psychotic and nonpsychotic diagnoses. In SZ patients, neuroimaging studies support the assumption for somewhat greater gray matter reduction in cingulate cortex (anterior and posterior), dorsolateral prefrontal and frontotemporal cortex, limbic structures (hippocampus), and basal ganglia (striatum). However, the magnitude of these structural changes is dependent on duration and severity of substance use, and at least in some of DD subjects, preexisting brain abnormalities are less pronounced than in pure SZ ones, which corresponds to better social and cognitive functioning and in general to lower neurodevelopmental and/or genetic pathological diathesis. As most studies on SZ also included schizoaffective diagnoses, thus probably enrolling a significant proportion of bipolar DD subjects, it is reasonable to compare their findings to what is reported by studies with “pure” bipolar patients. In doing so, the dorsolateral prefrontal, cingular, and insular cortices emerge as commonly affected areas in both SZ and BD. Taken together, these findings may implicate a shared endophenotypic (i.e., transdiagnostic) disruption of brain areas involved in executive functioning, emotional processing, and social cognition, which renders affected individuals susceptible to both mental disorder and substance misuse. Notably, gray matter loss in the anterior insula and dorsal part of the anterior cingular cortex has also been emphasized as a transdiagnostic finding in psychiatric patients in a nuber of recent studies (100, 101).

In comorbidity of anxiety and stress-related disorders (PTSD), as well as externalizing disorders with substance misuse, a common neuroimaging finding is the reduced volume of limbic structures (n. accumbens, hippocampus, amygdala). However, whether this reflects an underlying neuropathological characteristic predisposing to both specific behavioral symptoms and drug addiction or is a secondary effect of self-medication substance misuse on brain reward circuitry remains to be clarified.

## Author Contributions

Data review and analysis and manuscript preparation are performed by the same author (KS).

## Funding

This paper has been supported by a research grant form Medical University Pleven, Bulgaria.

## Conflict of Interest

The author declares that the research was conducted in the absence of any commercial or financial relationships that could be construed as a potential conflict of interest. The reviewer VN declared a past co-authorship with one of the authors KS to the handling Editor.
